# The ultrasonically treated nanoliposomes containing PCV2 DNA vaccine expressing gC1qR binding site mutant Cap is efficient in mice

**DOI:** 10.3389/fmicb.2022.1077026

**Published:** 2023-01-11

**Authors:** Qian Du, Tengfei Shi, Huaxin Wang, Changlei Zhu, Nan Yang, Dewen Tong, Yong Huang

**Affiliations:** ^1^College of Veterinary Medicine, Northwest A&F University, Xianyang, China; ^2^Engineering Research Center of Efficient New Vaccines for Animals, Ministry of Education, Xianyang, China

**Keywords:** porcine circovirus type 2, DNA vaccine, gC1qR binding site mutant Cap, expression enhancement, nanoliposome

## Abstract

Nowadays, vaccines are broadly used to prevent porcine circovirus type 2 (PCV2) infection-induced expenditures, but the virus is still spreading among pigs. The current PCV2 vaccines all rely on the immunogenicity of Cap, yet our previous studies found that Cap is also the major component mediating the PCV2 infection-induced immune suppression through its interaction with host gC1qR. Thereby, new vaccines are still necessary for PCV2 prevention and control. In this study, we constructed a new PCV2 DNA vaccine expressing the gC1qR binding site mutant Cap. We introduced the Intron A and WPRE elements into the vector to improve the Cap expression level, and fused the IL-2 secretory signal peptides to the N-terminal of Cap to mediate the secretion of Cap. We also screened and selected chemokines CXCL12, CCL22, and CCL25 to migrate dendritic cells. In addition, we contained the vectors with PEI and then ultrasonic them into nano size to enhance the entrance of the vectors. Finally, the animal experiments showed that the new PCV2 DNA vaccine expressing the gC1qR binding site mutant Cap could induce stronger humoral and cellular immune responses than the PCV2 DNA vaccine expressing the wild-type Cap and the non-ultrasonic treated PCV2 DNA vaccine in mice, and protect the mice from PCV2 infection and lung lesions. The results indicate the new PCV2 DNA vaccine expressing the gC1qR binding site mutant Cap has a certain development value, and provide new insight into the development of novel PCV2 vaccines.

## Introduction

Porcine circovirus type 2 (PCV2) is the primary pathogen of porcine circovirus-associated diseases (PCVAD) which is a major problem facing the global swine industry (Jimenez-Arriagada et al., 2022). PCV2 is widely prevalent in various pig-raising countries of the world, ranging from household pig farms to standardized pig farms (Li et al., 2021; Noh et al., 2022). On PCV2-positive farms, pigs of all ages can be infected by PCV2 and persistently carry the virus, but the most commonly diseased pigs are 6–8 weeks old. The pathogenesis of PCV2 is mainly attributed to the fact that PCV2 primarily attacks the host’s immune cells to suppress the immune system of infected piglets, leading to secondary or concurrent infection with multiple pathogens (Hansen et al., 2010; Sahoo et al., 2022). Since the commercial vaccines come into the market in 2006, the PCV2-induced clinical signs are effectively reduced, and pork production is improved (Guo et al., 2022). But for more than a decade, PCV2 has still been an epidemic in the pig herds of the world’s major pig-raising countries.

Porcine circovirus type 2 genome contains two major open reading frames, coding the replicase of the virus and the only capsid protein (Cap), respectively (Doan et al., 2022; Yang et al., 2022b). Cap is demonstrated to be the main source of the antigenic epitope, and all the genetic engineering vaccines for PCV2 are constructed using Cap (Nawagitgul et al., 2000). However, our previous studies found that Cap is also the most important pathogenic component of PCV2 during infection, the interaction of Cap with host protein gC1qR mediates the high production of IL-10 (Du et al., 2016), suppression of IL-12 production and Th1 cell activation (Du et al., 2018), as well as suppression of the type I interferon production. Besides, we found that Cap interacts with gC1qR by the motif 24RRR26 of the N-terminal (Wang et al., 2021). So, we speculate that mutation of the motif of Cap may enhance the effect of PCV2 vaccines. Some of the PCV2 vaccines are constructed with truncated Cap without the 1-41 aa nuclear localization signal (NLS) peptide, but recent studies clarify that the NLS peptide is critical for PCV2 particle assembly and may play roles in the immunity (Mo et al., 2019; Yu et al., 2021). Thereby, continuing to improve these vaccines or develop new vaccines so that they do not suppress the host immunity will be a new direction for PCV2 vaccine development. On the other hand, the smallest single-strand circular DNA genome of PCV2 makes the highest variation of PCV2 genotypes (Lefebvre et al., 2009; Hou et al., 2019; Yuan et al., 2022). Although the commercial PCV2 vaccines have been proven to remain effective in protecting pigs against the most spread PCV2a, PCV2b, and PCV2d strains, the emerging new PCV2 strains may break through vaccine protection (Guo et al., 2022). Thus, a vaccine platform that can be fast and easily operated is important to chase the evolutionary PCV2 strains.

Immunization with nucleic acids has received considerable attention in the field of new-generation vaccines that are easy to produce, safe, and can stimulate both humoral and cellular immune responses. The global pandemic of severe acute respiratory syndrome coronavirus 2 (SARS-CoV-2) in 2020 promotes the development of nucleic acid vaccines and further proves their effectiveness of them (Lederer et al., 2020; Polack et al., 2020; Widge et al., 2020). DNA vaccine delivers immunogenic antigens to the host’s cells by using DNA plasmids as a vector, which is more stable and cheaper to obtain than RNA vaccine. There are several studies have tried to research PCV2 DNA vaccines, they inserted the PCV2 ORF2 gene into the eukaryotic expression vector pEGFP-N1 or pcDNA3.1, and then found the recombinant plasmids could induce an anti-PCV2 immune response and protect BALB/c mice or Kunming mice from PCV2 infection (Sylla et al., 2014; Wang et al., 2015a). However, the vaccine efficacy was limited, and more importantly, the pathogenicity of Cap was not been fully considered. Therefore, it is still necessary to develop an efficient PCV2 DNA vaccine that does not have the ability to suppress the host immune response.

The undivided messenger RNA is exported to the cytoplasm with very low efficiency, but some viruses try to effectively export the undivided RNA to the cytoplasm. This is achieved through the synergistic or post-transcriptional binding of *cis*-acting elements with viruses or cytokines. WPRE is such a *cis*-acting element (Donello et al., 1998). If the WPRE element is placed downstream of the sense direction of the transgenic sequence, the expression efficiency can be improved by 5∼8 times. WPRE has widely been used to enhance transgene expression or virus titers in a series of vector contexts (Schwenter et al., 2003). Introns are the main part of non-coding sequences, which can increase the coding ability of the genome through alternative splicing and can also regulate gene expression in a variety of ways. It has been proved that some introns can enhance the expression of transgenes in different mammalian cell lines. Some intron elements can enhance transgenic expression in mammalian cell systems. For example, intron A of human cytomegalovirus (CMV) plays an active role in gene expression in transient and stable transfection of monkey kidney cells, human foreskin fibroblasts, HeLa cells, CHO-K1, and HEK 293 cells (Chapman et al., 1991).

In this present study, we constructed a plasmid expressing gC1qR binding site mutant Cap, and inserted the Intron A and Woodchuck Hepatitis Virus Posttranscriptional Regulatory Element (WPRE) into the plasmid to enhance the expression of mutant Cap. In order to make the expressed mutant Cap secret out of the cells and then can be effectively recognized by antigen-presenting cells, we fused the IL-2 secretory signal peptides to the N-terminus of mutant Cap. Moreover, we constructed vectors expressing chemokines CXCL12, CCL22, and CCL25 to further enhance the immune response of the host. To improve the entrance efficiency of the vectors into cells, we prepared the polyethylenimine (PEI) packaged vectors into nanoparticles. Finally, we conducted animal experiments to evaluate the efficacy of this PCV2 DNA vaccine. The results will provide a reference for the development and application of the PCV2 DNA vaccine.

## Materials and methods

### Ethics statement

The animal experiments were approved by the Institutional Animal Care and Use Committee (IACUC) of Northwest A&F University, China (permit number: 20201125) and were performed according to the Animal Ethics Procedures and Guidelines of the People’s Republic of China. No other specific permissions were required for these activities. This study did not involve endangered or protected species.

### Virus, vectors, and reagents

Porcine circovirus type 2 strain (GenBank: MH492006) was stored in our lab and propagated in PK-15 cells. The viral copies were measured by quantitative PCR. The vector pUC-A-Cap-WPRE containing the *intron A* (nucleotides 1265–2088, GenBank: M60321) and *WPRE* (nucleotides 1095–1670, GenBank: M18752) genes were constructed previously in our lab. The plasmid pVAX1 was purchased from Invitrogen. Polyethylenimine (PEI) was purchased from Sigma.

### Plasmids construction

The *cap* gene was firstly amplified from the genome of PCV2, and cloned into the pVAX1 plasmid, named pVAX-Cap. Then the sequence of the gC1qR binding site on the *cap* gene was mutated, named pVAX-Cap^mut^. Briefly, the overlap primers were designed in which the arginine residues (24 to 26) encoding nucleotides of Cap were replaced by alanine (AAA)-encoding nucleotides, the two sequences both containing the mutations were amplified by PCR, respectively. Then the full-length Cap was amplified using the two sequences as templates by PCR. The primers were 5′-CTTGGTACCGAGCTCGGATCCGCCACCATGACGTATC CAAGGAGGCGT-3′, 5′-CCACACTGGACTAGTGGATCCTTAG GGTTTAAGTGGGGGGTCT-3′, 5′-TATAACGCAGCAGCAAGCA TAAAT-3′, and 5′-AAATGCCATTCGTCGTCGTCCAGC-3′. Further, the gene of IL-2 secretory signal peptides (MYKMQLLCCIALTLALMANG, Genbank: NM_213861) was fused to the 5′ end of the wild-type *cap* gene or the mutant *cap* gene using homologous recombination, named pVAX-spCap and pVAX-spCap^mut^, and then the *intron A* and *WPRE* genes were inserted before or after the *cap* genes, respectively, named pVAX-A-spCap-W and pVAX-A-spCap^mut^-W.

The *cxcl12* (GenBank: NM_001012477), *ccl22* (GenBank: NM_009137), and *ccl25* (GenBank: NM_009138) genes were amplified from the peripheral blood mononuclear cells of mice, and cloned into pVAX1 plasmid, respectively. Then the plasmids expressing CXCL12, CCL22, and CCL25 were mixed equally for further use.

### Quantitative PCR

The pVAX1, pVAX-Cap, pVAX-Cap^mut^, pVAX-A-spCap-W, and pVAX-A-spCap^mut^-W were transfected into HEK 293T cells for 12 h, 24 h, and 48 h, respectively. The total RNAs of the cells were isolated using Trizol, and reverse transcripted using MLV. The mRNA levels of Cap were quantified by a Bio-Rad IQ5 real-time PCR system. The primers were Cap mRNA level:Cap-F TTGAATGTGGAGCTCCTAGAT, Cap-R GCAAGGTACTCACAGCAGTAGACA; β-actin-F GGACTTCGAG CAGGAGATGG, β-actin-R AGGAAGGAGGGCTGGAAGAG; viral copies: VD-F 5′-ATAACCCAGCCCTTCTCCTACC-3′, VD-R 5′-GGCCTACGTGGTCTACATTTCC-3′.

### Western blot

The pVAX-Cap, pVAX-Cap^mut^, pVAX-A-spCap-W, and pVAX-A-spCap^mut^-W transfected into 293T cells for 48 h, and then the cells were lysed with RIPA. The expression levels of Cap were detected using a rabbit anti-PCV2 serum obtained in our lab. The detailed steps are as described previously (Wu et al., 2019).

### Preparation of ultrasonic PEI containing plasmids

The plasmids were dissolved into 100 μL PBS to a final concentration of 500 ng/μL, and then gently mixed with 100 μL PEI with a concentration of 0.1 g/L. The mixtures were ultrasonicated in the ice bath for 2, 4, 6, 8, 10, and 12 min, setting as 150 w, 640 Hz, and 5 s break. The average particle sizes of the ultrasonic mixtures were measured by a nanometer laser particle size analyzer. The transfection efficiency of the ultrasonic mixtures and the non-ultrasonic mixtures were measured by GFP expression, that 1/10 amount of pEGFP-N1 plasmids were added into the mixtures.

### Animal experiments design

Seventy-two 6-week-old BALB/c mice were randomly divided into 6 groups with 12 mice in each group. One group mouse was set as control (Ctrl), one was set as challenge control (CC), and the other four groups were injected with 100 μg of pVAX-A-spCap-W, pVAX-A-spCap^mut^-W, Nano-PEI containing pVAX-A-spCap^mut^-W (NP-pVAX-A-spCap^mut^-W), and Nano-PEI containing pVAX-A-spCap^mut^-W and 1/10 amount of chemokine expressing plasmids (NP-pVAX-A-spCap^mut^-W-C). The mice of the four injection groups were injected on legs twice at 0 day and 7 days post-vaccination. At 28 days post-vaccination, 6 mice in each group were euthanized for the spleen lymphocyte proliferation assay, while the rest of the challenge control group and the injection groups of mice were challenged oronasally with 1 × 10^5^ copies of PCV2 for another 28 days. The Cap-specific antibody levels, PCV2 neutralizing antibody levels, and the serum viral loads were detected every 7 days post-vaccination. At 56 days post-vaccination, the mice were euthanized for further tests.

### Enzyme-linked immunosorbent assay

For the Cap-specific antibody detection, the prokaryotic expressed and purified Cap was coated on plates at 4°C overnight and then washed five times. The serum samples were incubated in wells of the plates for 1 h at room temperature, followed by washing five times. Then the Horseradish Peroxidase (HRP) conjugated goat anti-mouse IgG were incubated in wells for 30 min at 37°C. After the washing process, TMB substrates were added into the wells for 10 min in dark at 37°C. The OD_450nm_ values of each well were detected using a microplate reader after adding the stop solution.

For the IL-4 and IFN-γ detection, the supernatants of the cells were collected for detection using commercial ELISA kits (SEKM-0005 and SEKM-0031, Solarbio) according to the manufacturer’s instruction.

### PCV2 neutralization assay

The serums of the mice were serially diluted from 1:2 with medium and then incubated with PCV2 in the same amount at 37°C for 1 h. The viruses were infected into a 96-well plate containing PK-15 cells. At 72 h post-infection, the cells were incubated with rabbit anti-Cap serum at 4°C, and then with FITC-labeled goat anti-rabbit IgG. The viral amounts of these cells were detected and calculated for the titers of PCV2 neutralization antibodies (Li et al., 2017).

### Spleen lymphocyte proliferation assay

The spleen lymphocytes were isolated using a lymphocytes separation medium (P8860, Solarbio) according to the manufacturer’s instruction. The spleen lymphocytes were plated into 96-well plates, and stimulated by the prokaryotic expressed Cap, ConA, or medium for 68 h. Then the cells were treated with 3- (4,5)-dimethylthiahiazo (-z-y1)-3,5-di- phenytetrazoliumromide (MTT) in dark for 4 h. After the replacement of the medium to DMSO, the OD_570nm_ values were measured and used for calculation of the stimulation index as OD_570nm_ of Cap-stimulated cells/mean OD_570nm_ of non-stimulated cells.

### Histology analysis

The freshly collected lungs of the mice were fixed in formalin for 1 week. After being treated including dehydration, clearing, infiltration, and embedding in an automatic biological tissue dehydrator, the tissues were embedded in paraffin wax. The tissues were cut into 4 μm thick and taken to perform hematoxylin-eosin (H&E) staining using a glass slide automatic dyeing machine. The H&E sections were scanned using a Leica Aperio LV1.

### Statistical analysis

All data are presented as the mean ± SD. Statistical analyses were performed using GraphPad Prism 8 software, Comparisons between two groups were performed by an unpaired Student’s *t*-test, whereas data from multiple groups were analyzed by ANOVA. A value of *p* < 0.05 is considered significant.

## Results

### Construction of plasmids

**FIGURE 1:**
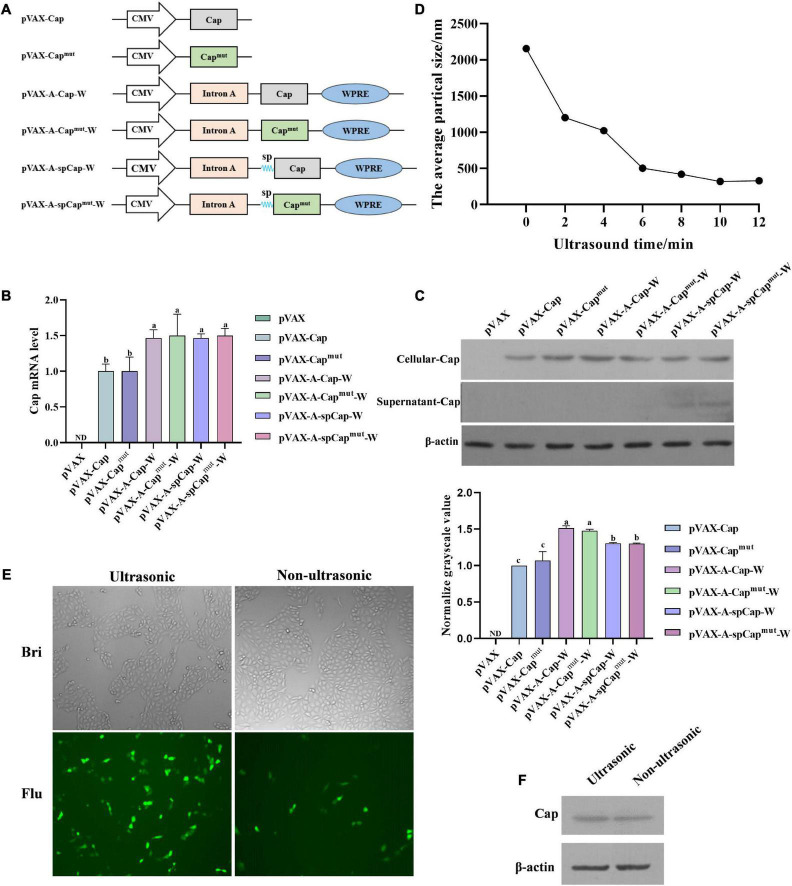
Construction of the plasmids and preparation of the porcine circovirus type 2 (PCV2) DNA vaccines. **(A)** Shematic structure of pVAX-Cap, pVAX-Cap^mut^, pVAX-A-Cap-W, pVAX-A-Cap^mut^-W, pVAX-A-spCap-W, pVAX-A-spCap^mut^-W, and the plasmids expressing CXCL12, CCL22, and CCL25. Human cytomegalovirus (CMV) represents the promoter of the vectors, sp represents the IL-2 secretory signal peptides. **(B)** The pVAX-Cap, pVAX-Cap^mut^, pVAX-A-Cap-W, pVAX-A-Cap^mut^-W, pVAX-A-spCap-W, and pVAX-A-spCap^mut^-W plasmids were transfected into 293T cells with equal amount for 48 h, then the mRNA levels of Cap were measured by qPCR. **(C)** The pVAX-Cap, pVAX-Cap^mut^, pVAX-A-Cap-W, pVAX-A-Cap^mut^-W, pVAX-A-spCap-W, and pVAX-A-spCap^mut^-W plasmids were transfected into 293T cells with equal amount for 48 h, the expression levels of Cap in the transfected cells and in the supernatants of these cells were examined by western blot. **(D)** The polyethylenimine (PEI) containing plasmids were treated with ultrasonic for 2, 4, 6, 8, 10, and 12 min, respectively. The particle sizes of the treated mixtures were analyzed. **(E,F)** The ultrasonic PEI containing pVAX-Cap and 1/10 pEGFP-N1 and the non-ultrasonic PEI containing pVAX-Cap and 1/10 pEGFP-N1 were transfected into PK-15 cells for 48 h, the transfection efficiency was analyzed by fluorescence microscopy **(E)** and western blot **(F)**. ND means not detected, the same small letters (a and b) represent no significant difference among the groups, and different small letters represent significant differences among the groups in **(B)**.

In order to develop a new PCV2 DNA vaccine platform, and investigate whether the gC1qR binding site mutant PCV2 Cap has better immunogenicity than the wild-type Cap, we first amplified the *cap* gene from the genome of PCV2, and cloned it into the pVAX1 plasmid, named pVAX-Cap (Figure 1A). Then we mutated the sequence of gC1qR binding site on the *cap* gene using site-specific mutagenesis, named pVAX-Cap^mut^ (Figure 1A). Further, we fused the gene of IL-2 secretory signal peptides to the 5′ end of the wild-type *cap* gene or the mutant *cap* gene using homologous recombination, named pVAX-spCap and pVAX-spCap^mut^, and then inserted the *intron A* and *WPRE* genes before or after the *cap* genes, respectively, named pVAX-A-spCap-W and pVAX-A-spCap^mut^-W (Figure 1A). To further enhance the PCV2 DNA vaccine-induced immune response, we screened the chemokines that mainly target antigen-presenting cells and selected CXCL12, CCL22, and CCL25 for further study. We amplified the *cxcl12, ccl22*, and *ccl25* genes from the peripheral blood mononuclear cells of mice, and cloned them into pVAX1 plasmid, respectively. Then we mixed the plasmids expressing CXCL12, CCL22, and CCL25 equally (Figure 1A).

### Intron A and WPRE elements enhance Cap expression of the plasmids, and IL-2 secretory signal peptides mediate the secretion of Cap out of the transfected cells

To figure out if the gC1qR binding site mutation and the Intron A, WPRE, and IL-2 secretory signal peptides elements can alter the expression of Cap, we transfected 293T cells with pVAX1, pVAX-Cap, pVAX-Cap^mut^, pVAX-A-Cap-W, pVAX-A-Cap^mut^-W, pVAX-A-spCap-W, and pVAX-A-spCap^mut^-W for 48 h, respectively. The mRNA expression levels results showed that the mutation of gC1qR binding site on Cap did not significantly alter the expression levels of Cap between the pVAX-Cap transfected cells and the pVAX-Cap^mut^ transfected cells, or among the pVAX-A-Cap-W transfected cells, pVAX-A-Cap^mut^-W transfected cells, pVAX-A-spCap-W transfected cells, and pVAX-A-spCap^mut^-W transfected cells (Figure 1B). While, the pVAX-A-Cap-W transfected cells, pVAX-A-Cap^mut^-W transfected cells, pVAX-A-spCap-W transfected cells, and the pVAX-A-spCap^mut^-W transfected cells expressed significantly higher levels of Cap mRNA than the pVAX-Cap transfected cells and the pVAX-Cap^mut^ transfected cells. Besides, the IL-2 secretory signal peptides did not significantly alter the expression of Cap that the levels of the pVAX-A-spCap-W transfected cells and the pVAX-A-spCap^mut^-W transfected cells were same as that of the pVAX-A-Cap-W transfected cells and the pVAX-A-Cap^mut^-W transfected cells (Figure 1B). Meanwhile, the western blot results of the transfected cells for 48 h showed that Cap expression could be detected in the lysis of all the transfected cells, while only in the supernatants of the pVAX-A-spCap-W transfected cells and the pVAX-A-spCap^mut^-W transfected cells, but not in the pVAX-Cap transfected cells, pVAX-Cap^mut^ transfected cells, pVAX-A-Cap-W transfected cells, and pVAX-A-Cap^mut^-W transfected cells (Figure 1C). These results indicate that the gC1qR binding site mutation does not influence the Cap expression of the plasmids, Intron A and WPRE enhance the expression level of Cap, and the IL-2 secretory signal peptides mediate the secretion of Cap out of the cells.

### Ultrasonic treatment of the PEI containing plasmids further enhances the Cap expression in transfected cells

Further, to figure out whether the ultrasonic treatment of the PEI containing plasmids can enhance their efficiency in transfecting cells, we contained pVAX-Cap and 1/10 amount of pEGFP-N1 with PEI, then treated with ultrasonic for 2, 4, 6, 8, 10, and 12 min, respectively. The results showed that the particle sizes were reduced along with the ultrasonic time to nano size (Figure 1D). Then we transfected PK-15 cells with the ultrasonic PEI containing pVAX-Cap and pEGFP-N1 and the non-ultrasonic PEI containing pVAX-Cap and pEGFP-N1 for 48 h, respectively. The results showed that the ultrasonic treatment visibly enhanced the expression levels of the plasmids (Figure 1E). The Cap expression level results showed that the ultrasonic PEI containing pVAX-Cap transfected cells expressed a higher level of Cap than the non-ultrasonic PEI containing pVAX-Cap transfected cells (Figure 1F). These results show that containing of the plasmids using PEI and then treated using ultrasonic can significantly enhance the expression levels of proteins encoding by the plasmids.

### The PCV2 DNA vaccines do not obviously affect the temperature and weight of mice

**FIGURE 2:**
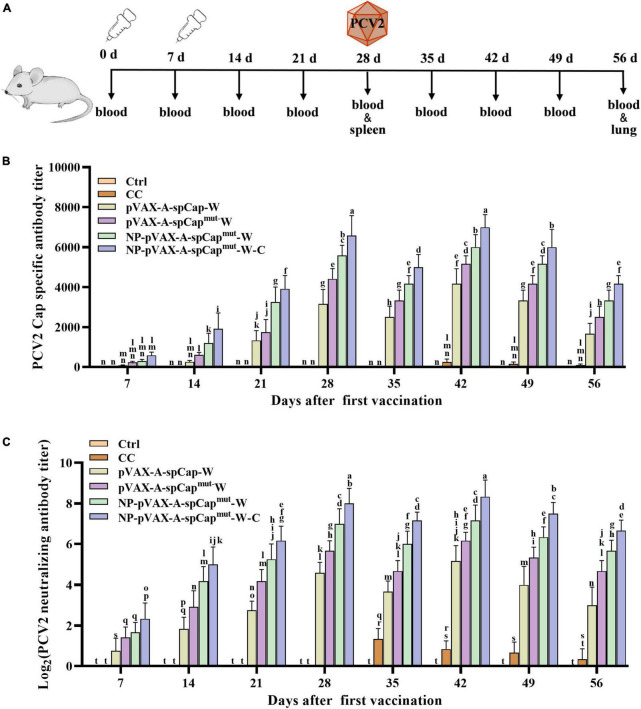
The gC1qR binding site mutation Cap induces higher humoral immune response than the wild-type Cap in mice. **(A)** Vaccination schedule and blood collection, spleen collection, lung collection, and challenge times. **(B)** The serum samples of the vaccinated mice were assayed for porcine circovirus type 2 (PCV2) Cap-specific antibody levels by enzyme-linked immunosorbent assay (ELISA). **(C)** PCV2 were incubated with the serum samples of the vaccinated mice, and then infected to PK-15 cells for 72 h. The PCV2 levels were detected, and the PCV2 neutralizing antibody levels were calculated. The same small letters (a∼n) represent no significant difference among the groups, and different small letters represent significant differences among the groups in **(B,C)**.

To figure out the influence of the PCV2 DNA vaccines on the physiological indicators, we randomly divided seventy-twos 6-week-old BALB/c mice into 6 groups with 12 mice in each group. We set one of the groups of mice as control (Ctrl), another group as challenge control (CC), and injected the other four groups of mice with 100 μg of pVAX-A-spCap-W, pVAX-A-spCap^mut^-W, Nano-PEI containing pVAX-A-spCap^mut^-W (NP-pVAX-A-spCap^mut^-W), and Nano-PEI containing pVAX-A-spCap^mut^-W and 1/10 amount of chemokine expressing plasmids (NP-pVAX-A-spCap^mut^-W-C), respectively. The mice of the pVAX-A-spCap-W, pVAX-A-spCap^mut^-W, NP-pVAX-A-spCap^mut^-W, and NP-pVAX-A-spCap^mut^-W-C groups were vaccinated twice at 0 day and 7 days post-vaccination, and then challenged with PCV2 at 28 days post-vaccination (Figure 2A). The temperatures and body weights of all mice were detected every 7 days. The results showed that all mice maintained to the normal temperature before the PCV2 challenge at 28 days post-vaccination. After challenge of PCV2, the temperatures of the CC group mice were increased higher than the pVAX-A-spCap-W, pVAX-A-spCap^mut^-W, NP-pVAX-A-spCap^mut^-W, and NP-pVAX-A-spCap^mut^-W-C group mice at 35 days post-vaccination and 42 days post-vaccination, and then recovered to the normal temperature (data not shown). However, the average weight gains of all groups were not significantly changed (data not shown). These results show that the prepared PCV2 DNA vaccines have little effect on the physiological indicators of mice.

### gC1qR binding site mutation Cap induces higher humoral immune response than wild-type Cap in mice

Furthermore, to test the ability of pVAX-A-spCap-W, pVAX-A-spCap^mut^-W, NP-pVAX-A-spCap^mut^-W, and NP-pVAX-A-spCap^mut^-W-C to stimulate humoral immunity, we detected the antibody levels in the serum of the mice every 7 days. The Cap-specific antibody results showed that the Cap-specific antibodies could not be detected in the serum of the Ctrl group mice period all time points and the CC group mice before 35 days post-vaccination, while the Cap-specific antibodies were detectable since 7 days post-vaccination in the serum of the pVAX-A-spCap-W, pVAX-A-spCap^mut^-W, NP-pVAX-A-spCap^mut^-W, and NP-pVAX-A-spCap^mut^-W-C groups mice (Figure 2B). Detailly, the Cap-specific antibody levels of the pVAX-A-spCap-W, pVAX-A-spCap^mut^-W, NP-pVAX-A-spCap^mut^-W, and NP-pVAX-A-spCap^mut^-W-C groups mice were increased from 7 days post-vaccination to 28 days post-vaccination, reduced at 35 days post-vaccination, and then increased to the peak at 42 days post-vaccination, subsequently decreased (Figure 2B). Comparing to the Cap-specific antibody levels of the mice in the wild-type Cap expressing group pVAX-A-spCap-W, the Cap-specific antibody levels were significantly higher of the mice in the gC1qR binding site mutant Cap expressing groups pVAX-A-spCap^mut^-W, NP-pVAX-A-spCap^mut^-W, and NP-pVAX-A-spCap^mut^-W-C at all time points (Figure 2B). The results also showed that the Cap-specific antibody levels of the mice in the ultrasonic treatment of the PEI containing plasmids groups NP-pVAX-A-spCap^mut^-W and NP-pVAX-A-spCap^mut^-W-C were higher than the mice in the non-treatment groups pVAX-A-spCap-W and pVAX-A-spCap^mut^-W (Figure 2B). In addition, the chemokines further increased the Cap-specific antibody levels of the mice in the NP-pVAX-A-spCap^mut^-W-C group than the mice in the NP-pVAX-A-spCap^mut^-W group (Figure 2B). The PCV2 neutralization antibody levels showed the similar results as the Cap-specific antibody levels (Figure 2C). These results demonstrate that the gC1qR binding site mutant Cap could induce higher level of humoral immune response than the wild-type Cap in mice, and the ultrasonic treatment of the PEI containment and the chemokines addition further improve the ability of the PCV2 DNA vaccines to stimulate the host humoral immune response to produce antibodies.

### gC1qR binding site mutation Cap also induces more cellular immune response than wild-type Cap in mice

**FIGURE 3:**
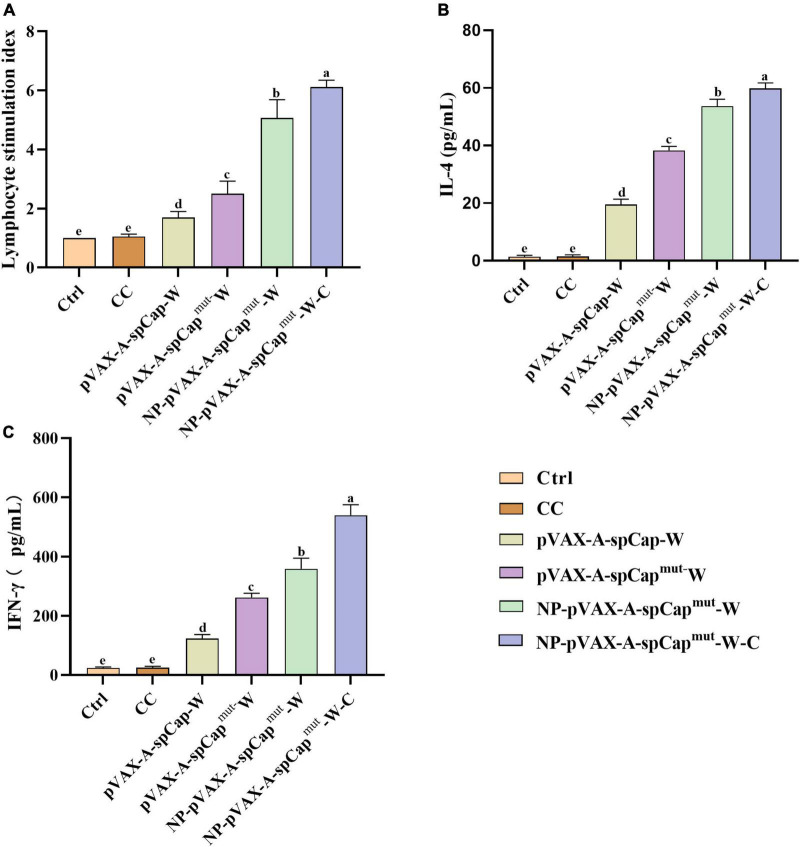
The gC1qR binding site mutation Cap induces higher cellular immune response than the wild-type Cap in mice. At 28 days post-vaccination, half of the mice in each group were euthanized, and then the spleen lymphocytes were isolated for further assays. **(A)** The spleen lymphocytes of mice in each group were plated into 96-well plates, and stimulated by the prokaryotic expressed Cap, ConA as positive control, or medium as negative control for 68 h. Then the proliferation level of spleen lymphocytes in mice was detected by 3- (4,5)-dimethylthiahiazo (-z-y1)-3,5-di- phenytetrazoliumromide (MTT) assay. **(B,C)** The IL-4 **(B)** and IFN-γ **(C)** levels in the supernatant of these lymphocytes stimulated by the prokaryotic expressed Cap were detected by enzyme-linked immunosorbent assay (ELISA). The same small letters (a∼e) represent no significant difference among the groups, and different small letters represent significant differences among the groups in **(A–C)**.

To further examine the cellular immune response of the mice, we harvested the spleen lymphocytes of three mice in each group on 28 days post-vaccination. The lymphocyte proliferation assay results showed that the lymphocyte proliferation levels of the pVAX-A-spCap-W, pVAX-A-spCap^mut^-W, NP-pVAX-A-spCap^mut^-W, and NP-pVAX-A-spCap^mut^-W-C groups mice were higher than the Ctrl and CC groups mice in general (Figure 3A). The lymphocyte proliferation levels of the gC1qR binding site mutant Cap expressing pVAX-A-spCap^mut^-W, NP-pVAX-A-spCap^mut^-W, and NP-pVAX-A-spCap^mut^-W-C groups mice were higher than the wild-type Cap expressing pVAX-A-spCap-W group mice (Figure 3A). Meanwhile, the lymphocyte proliferation levels of the NP-pVAX-A-spCap^mut^-W and NP-pVAX-A-spCap^mut^-W-C groups mice were higher than the pVAX-A-spCap^mut^-W group mice, but they were at the similar level in the NP-pVAX-A-spCap^mut^-W and NP-pVAX-A-spCap^mut^-W-C groups mice (Figure 3A). We also detected the IL-4 and IFN-γ production levels of the spleen lymphocytes from mice, and the results showed that the IL-4 and IFN-γ production levels of the pVAX-A-spCap-W, pVAX-A-spCap^mut^-W, NP-pVAX-A-spCap^mut^-W, and NP-pVAX-A-spCap^mut^-W-C groups mice were higher than the Ctrl and CC groups mice, they were also higher in the gC1qR binding site mutant Cap expressing pVAX-A-spCap^mut^-W, NP-pVAX-A-spCap^mut^-W, and NP-pVAX-A-spCap^mut^-W-C groups mice than in the wild-type Cap expressing pVAX-A-spCap-W group mice, but although the IFN-γ production levels of the NP-pVAX-A-spCap^mut^-W-C group mice was significantly higher than the NP-pVAX-A-spCap^mut^-W group mice, the IL-4 production levels of the NP-pVAX-A-spCap^mut^-W-C group mice was only slightly higher than the NP-pVAX-A-spCap^mut^-W group mice (Figures 3B, C). These results further confirm that the gC1qR binding site mutant Cap is better than the wild-type Cap in inducing cellular immune response of mice, meanwhile the ultrasonic treatment of the PEI containment and the chemokines addition enhance the immune response.

### The serum viral loads and histopathological lesions are lighter in the gC1qR binding site mutant Cap group mice than in the wild-type Cap group mice

**FIGURE 4:**
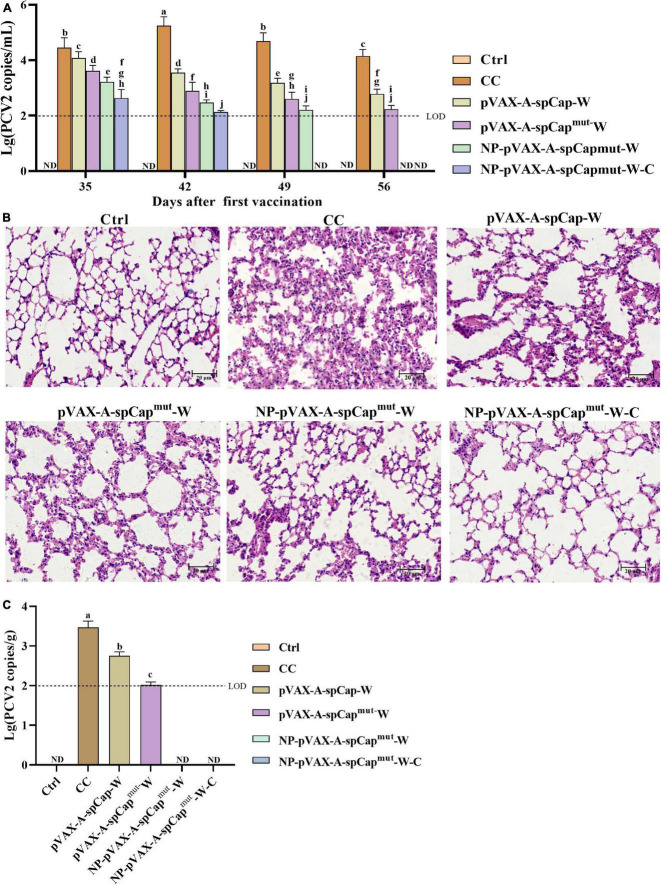
The gC1qR binding site mutation Cap is more efficient in protecting mice from porcine circovirus type 2 (PCV2) infection and the viral infection-induced lung lesions. **(A)** The PCV2 copy numbers in the serum of the different groups mice were measured by qPCR every 7 days after challenge. **(B)** At 56 days post-vaccination, all groups mice were euthanized, the lungs were made into paraffin tissue sections, stained with HE, and the pathological changes of the lung tissues were observed with a microscope. **(C)** The PCV2 copy numbers in the lungs of the different groups mice were measured by qPCR at 56 days post-vaccination. Bar = 20 μm. ND means not detected, and the same small letters (a∼j) represent no significant difference among the groups, and different small letters represent significant differences among the groups in **(A)**.

To make clear the ability of pVAX-A-spCap-W, pVAX-A-spCap^mut^-W, NP-pVAX-A-spCap^mut^-W, and NP-pVAX-A-spCap^mut^-W-C in protecting mice from the infection of PCV2, we detected the PCV2 DNA copies in the serum of the mice every 7 days since PCV2 challenge at 28 days post-vaccination. The results showed that the serum PCV2 loads of the pVAX-A-spCap-W, pVAX-A-spCap^mut^-W, NP-pVAX-A-spCap^mut^-W, and NP-pVAX-A-spCap^mut^-W-C groups mice were significantly lower than the CC group mice from 35 days post-vaccination to 56 days post-vaccination (Figure 4A). The serum viral loads of the pVAX-A-spCap^mut^-W, NP-pVAX-A-spCap^mut^-W, and NP-pVAX-A-spCap^mut^-W-C groups mice were significantly lower than the pVAX-A-spCap-W group mice, and the serum viral loads of the NP-pVAX-A-spCap^mut^-W and NP-pVAX-A-spCap^mut^-W-C groups mice were lower than the pVAX-A-spCap^mut^-W group mice (Figure 4A). The viral loads of the NP-pVAX-A-spCap^mut^-W-C group mice became undetectable since 49 days post-vaccination, while the viral loads of the NP-pVAX-A-spCap^mut^-W group mice were under the detection limits at 56 days post-vaccination (Figure 4A). We also checked the pathogenic changes of the lungs of these mice, and the results showed that the lungs of the CC group mice had obvious lesions, with infiltrated by inflammatory cells, thickened of the alveolar wall, and shed of the alveolar epithelial cells (Figure 4B). The lungs of the pVAX-A-spCap-W and pVAX-A-spCap^mut^-W groups mice still had lesions, although better than the lungs of the CC group mice (Figure 4B). No visible lesions were found in the lungs of the NP-pVAX-A-spCap^mut^-W, and NP-pVAX-A-spCap^mut^-W-C groups mice (Figure 4B). The PCV2 loads of the lungs were measured by qPCR, and showed the similar results of the pathogenic lesions (Figure 4C). These results show that vaccination of the ultrasonic PEI containing PCV2 DNA vaccines can effectively protect mice from PCV2 infection.

## Discussion

Porcine circovirus type 2 causes reproductive disorders in sows, postweaning multisystemic wasting syndrome, porcine dermatitis and nephropathy syndrome, congenital tremor in piglets, porcine respiratory syndrome, necrotizing interstitial pneumonia, etc. (Liu et al., 2017). More importantly, PCV2 induces immunosuppression in pigs, thus making pigs susceptible to various pathogens, and causing huge economic losses to the pig industry (Yang et al., 2018, 2022a; Sidler et al., 2020). Vaccination has become an important means of preventing and controlling PCVAD, but it has been reported that the existing commercial vaccines cannot completely resist the invasion of PCV2 (Oh et al., 2022). Therefore, new vaccines are needed to meet the challenges posed by PCV2. DNA vaccines are quick to prepare, easy to produce, stable in storage, and induce longer immune protection in the host (Volokhov et al., 2022; Wang et al., 2022a). Meanwhile, the antigen of the DNA vaccines is expressed in the host cells, which can directly combine with MHC I and MHC II to trigger cellular immunity and humoral immune response, thus the immune response will be triggered more quickly after vaccination (Volokhov et al., 2022). In this study, we constructed a new DNA vaccine platform for PCV2, the new PCV2 DNA vaccine was more efficient in entering the cells, expressing the PCV2 antigen Cap, and secreting the antigens out of cells for antigen-presenting cell capture and recognition, while the most important of all is that the mutation of the gC1qR binding site on Cap significantly enhanced humoral and cellular immune responses in vaccinated mice, which makes the new PCV2 DNA vaccine were better in protection the mice from PCV2 infection.

Cap is the only structural protein and the major antigen of PCV2 (Wang et al., 2019b). Nowadays, the PCV2 vaccines are all designed and produced based on Cap, including the previously reported studies on PCV2 DNA vaccines (Wang et al., 2015b). However, it has been found that PCV2 Cap interacts with multiple host proteins, including MKRN1, gC1qR, Par-4, NAP1, NPM1, and Hsp40 (Finsterbusch et al., 2009), and our previous studies showed that the interaction of Cap and host gC1qR plays a major role in the PCV2 infection-induced immunosuppression of host. We found that the PCV2 Cap and gC1qR interaction mediates the inhibition of the host type I interferons antiviral immune response (Wang et al., 2021, 2022b). PCV2 infection also suppresses IL12p40 expression and reduces host Th1 immunity through gC1qR-mediated activation of PI3K/Akt1 and p38 MAPK signaling pathways (Du et al., 2018). More importantly, blocking the interaction of Cap and gC1qR significantly prevents the suppression of host immune responses (Du et al., 2022). However, we also found that the interaction of Cap and gC1qR is important for the nuclear release of PCV2, thus the gC1qR binding site mutant PCV2 is difficult to proliferate to a high level (Wang et al., 2019a). Based on these facts, we constructed a PCV2 DNA vaccine expressing the gC1qR binding site mutant Cap. *In vitro* experiments showed that the mutation of the gC1qR binding site did not affect the expression levels of Cap. The animal experiment results demonstrated that the gC1qR binding site mutant Cap was more efficient in inducing humoral and cellular immune responses compared to the wild-type Cap. Accordingly, the serum PCV2 copies and the lung lesions were less in the gC1qR binding site mutant Cap vaccinated mice than that in the wild-type Cap vaccinated mice. All these results indicate that the interaction of Cap and gC1qR affects the immune response of PCV2 vaccines, and mutation of the gC1qR binding site on Cap significantly improves the immune protective effect of the PCV2 DNA vaccine. Another risk of the PCV2 DNA vaccine is that bio-safe. The previously reported studies on the PCV2 DNA vaccines found that the foreign gene did not recombine with the host genomes. In this study, we used the pVAX1 vector, which is specifically designed for use in the development of DNA vaccines, and we did not detect recombination in the tissues of the mice by PCR in this study (data not shown). However, more studies are still needed to confirm the safety of this PCV2 DNA vaccine.

The mRNA introns play important roles in the regulation of gene expression, while WPRE is a *cis*-acting RNA element that can significantly increase the mRNA expression level and translation efficiency when placed before the polyadenylation signal (Marcovich et al., 2022). In our previous study, we confirmed that the introduction of Intron A and WPRE into the recombinant adenovirus could significantly enhance the expression of Cap. In this work, we also constructed these Intron A and WPRE elements into the PCV2 DNA vaccine plasmids to improve the expression of the gC1qR binding site mutant Cap, and the results were confirmed in the transfected cells *in vitro*. In addition, to allow the expressed immunogen could be more easily to be recognized by antigen-presenting cells, we fused the IL-2 secretory signal peptides to the N-terminal of the gC1qR binding site mutant Cap. We also observed the secretion of the gC1qR binding site mutant Cap in the supernatant of the transfected cells *in vitro*. On the other hand, vaccine delivery systems have shown a significant impact on the protective effect of vaccines (Luo et al., 2022). Nanoliposomes are well-developed delivery systems, and they are convenient and quick to prepare, with low cost and high biological safety (Luo et al., 2022). PEI is cheap and efficient in delivering DNA into cells (Rahimi et al., 2022); thus, we employed PEI to encapsulate the PCV2 DNA vaccine. Furthermore, we treated the PEI and plasmid mixtures with ultrasonic into Nano size, that to further improve the capability of the cell entrance of the PCV2 DNA vaccines. We also screened the chemokines that have the ability to chemotactic DC cells, the major antigen-presenting cells, and we chose CXCL12, CCL22, and CCL25 (Olsnes et al., 2008; Gouwy et al., 2014). We believe that the co-expression of these chemokines can induce DC cells to migrate to the injection sites of the mice, thereby improving the recognition of expressed Cap by the host immune system, which enhances the effect of the PCV2 DNA vaccine. Finally, we confirmed all these improvements work as expected in the PCV2 DNA vaccine vaccinated mice.

In this study, we found and verified that the ultrasonic treated PEI containing gC1qR binding site mutant Cap can efficiently induce the immune responses of the mice and protect the vaccinated mice from PCV2 infection. The results provide new insight into the development of novel PCV2 vaccines. However, further studies are still needed to verify the immune effect of the new PCV2 DNA vaccine in pigs.

## Data availability statement

The original contributions presented in this study are included in this article/supplementary material, further inquiries can be directed to the corresponding authors.

## Ethics statement

The animal study was reviewed and approved by the Institutional Animal Care and Use Committee (IACUC) of Northwest A&F University, China.

## Author contributions

YH and QD contributed conception and design of the study. QD and TS performed the experiments and analyzed the data. HW, CZ, and NY participated in the animal experiments. QD and TS wrote the first draft of the manuscript. YH and DT contributed to the manuscript revision. All authors read and approved the submitted version.
